# Supplementary figures and images for: The Effect of Functional Fiber on Microbiota Composition in Different Intestinal Segments of Obese Mice
Source: Int J Mol Sci. 2021 Jun 18;22(12):6525. doi: 10.3390/ijms22126525 (PMC8234870; doi:10.3390/ijms22126525)

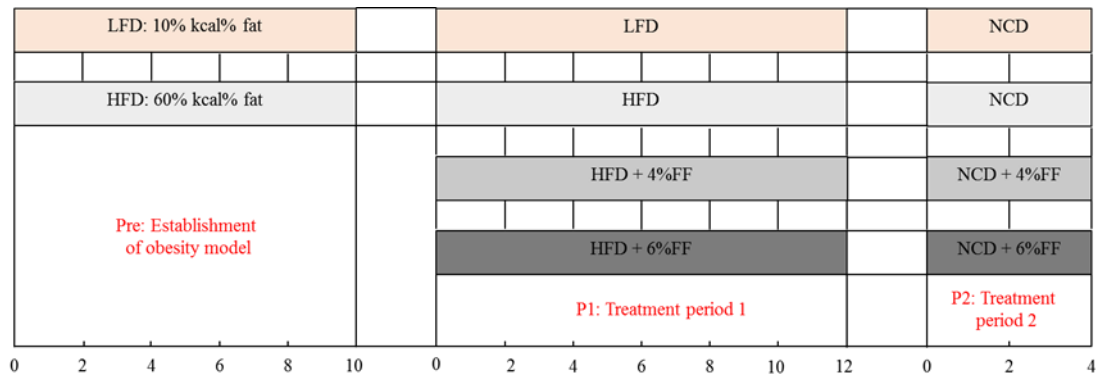

**Figure S1.** Overview of the study design.

Supplement: Supplementary file 1 [file ijms-22-06525-s001.zip › ijms-1216587-supplementary.pdf]
